# Protocol for creating a dataset of U.S. state alcohol-related firearm laws 2000–2022

**DOI:** 10.1371/journal.pone.0299248

**Published:** 2024-03-07

**Authors:** Diana Silver, Jin Yung Bae, James Macinko

**Affiliations:** 1 New York University School of Global Public Health, New York, NY, United States of America; 2 UCLA Fielding School of Public Health, University of California, Los Angeles, Los Angeles, CA, United States of America; PLoS ONE, UNITED STATES

## Abstract

Firearms are a major source of preventable morbidity and mortality in the United States, contributing to over 48,000 deaths in 2022 and generating societal costs in excess of $500 billion. A body of work has examined the relationship between US state level firearm laws and health outcomes, generally finding that some firearm regulations are associated with lower firearm-related mortality. Alcohol has been identified as an additional risk factor for both homicides and suicide and stronger state alcohol laws have been associated with lower rates of suicide. To date, there are no empirical studies that have investigated the impact of laws over a long period of time that target the intersection of alcohol and firearm. One reason for this may be because there is no existing dataset that includes the range of these state laws over time. This study describes the protocol for collecting, coding and operationalizing these legal data.

## Introduction

In the United States, firearms contributed to over 48,000 deaths in 2022. Over half (56%) of these deaths were suicides, which in 2022 reached an all-time high [1, 2].There is considerable evidence that alcohol contributes to firearm-related harms, including homicide, suicide, intimate partner violence, and unintentional deaths [3]. Firearm-related harms also incur considerable costs: medical care was estimated to have cost as much as $2.8 billion per year between 2006 and 2014 while other costs such as rehabilitation, long-term care, criminal justice, job loss, and mental health treatment were estimated at $174 billion in 2010 alone [4, 5]. A recent non- peer reviewed study puts the total U.S. costs of firearm violence at $557 billion or $1,698 per person per year [6]. These figures make clear the need to identify programs and policies that are effective in reducing such harms.

In the US, firearm purchases and use are primarily regulated at the state level. The main federal statutes related to firearms relate to background checks and do not mention alcohol [7]. As a consequence of the decentralized nature of firearm regulation, firearm-related laws vary substantially from state to state, so that some states have much stricter regulations on the purchase, permitting, carrying, and storage of firearms, while other states make it easier to acquire firearms and may even provide firearm owners greater legal protections. While homicide is the most frequently discussed firearm-related outcome, suicides represent more than half of the total number of firearm-related deaths and firearms are used in about half of suicide cases in the U.S [2]. Numerous studies have documented the strong association between excessive alcohol use and violence, on outcomes ranging from intimate partner violence, to assault, to suicide [8–15]. One study [16] reported an association between the strength of the state alcohol regulatory environment and homicide, and a systematic review of alcohol policies and suicide found evidence of protective effects of restrictive alcohol policies on state suicide rates [17]. Other studies point to the effect of alcohol outlet density [18, 19]. and alcohol advertising on violent crime [20].

Stronger state-level alcohol regulations are associated with reductions in binge and heavy drinking, fewer impaired driving-related motor-vehicle collisions (and subsequent deaths and injuries), and reductions in rates of interpersonal violence [21]. However, literature about the intersection of alcohol laws and firearm laws is scant. Branas et al’s [3] systematic review found only one study examining state laws that focus on the intersection between alcohol and firearms, that is, laws that explicitly restrict sales and/or possession of firearms to those convicted of alcohol related crimes, habitual users or intoxicated persons, or prohibit firearms in bars and restaurants. This study solely reported on results of a 50-state survey of these state laws in 2008, but did not test associations between the presence of these laws and firearm injury and fatality outcomes [4].

In an important study on the topic, Carr et al [22] conducted original legal research to identify several types of laws related to alcohol and firearms, but did not make these data publicly available. First, Carr identified laws that restrict sales, possession, usage, or concealed carry of a firearm among those found to be acutely intoxicated. According to that study, 26 states had at least one of these laws in 2008. Second, laws restricting firearm sales, possession, licensing, and concealed carry among those considered to be “habitual alcohol users”. In 2008, 20 states had at least one of these laws on the books. The authors note that the legislation largely leaves the terms “acutely intoxicated” and “habitual alcohol users” undefined [22]. Third, laws that prohibit having a loaded firearm where liquor is sold for consumption on premises. In 2010, Carr et al found that 12 states had such a law on the books. However, no publicly available dataset contains all of Carr et al’s laws, making it difficult to update them to the present day. The RAND state firearm law database [23] contains only two laws that reference alcohol-related firearm prohibited possessor laws (California) and for concealed weapons among those intoxicated (Idaho). The Boston University (BU) dataset of firearm laws [24] contains references to some of these laws, but simplifies them to include only two categories, both of which fit into Carr et al’s definition of restrictions among people with habitual alcohol problems and among people undergoing treatment for alcohol addiction. In addition to the categories of laws identified by Carr, some states include evidence of dangers associated with alcohol/drug use within their extreme risk protection orders (ERPO) laws as a cause for removing a gun. Based on this review and assessment of publicly-available firearm-related legal datasets, there appears to be a gap in identifying, describing, and assessing the impact of state laws that target both alcohol and firearms.

The proposed study seeks to expand the data available on alcohol-related firearm laws by creating a longitudinal, detailed dataset of all state level alcohol-related firearm laws, 2000–2022 and making these data public for use by researchers and policymakers.

## Materials and methods

The research team will identify and analyze the scope and content of state laws that explicitly target the intersection of alcohol and firearms. Using original legal research, we will identify the presence and effective dates of these alcohol-related firearm laws, and qualitatively assess the content of laws focused on restricting access, use, possession or carrying of a firearm 1) among individuals who are acutely intoxicated, 2) those who are considered to be “habitual” alcohol users, 3) those deemed to pose harm to themselves or others due to alcohol; and 4) venue-based restrictions on carrying a firearm where alcohol is served. Because we are simply coding publicly available legislative statutes which we retrieved, no IRB submission was necessary.

Our team, which includes experienced attorneys, will review and revise the existing coding scheme for each law based on the categories presented in Carr et al [22], and will additionally include the subset of ERPO laws that include alcohol, subject to additional modification based on results of the legal research. For each law, the dataset will include data for all 50 states from 2000 to 2022. Multiple variables will be coded for each law to reflect the presence of the corresponding law and multiple dimensions encompassing its nature and scope. These dimensions include, at a minimum: each law’s effective date; their scope, i.e., the definition of the population affected (including, if available, how alcohol use is determined); dimensions of enforceability such as inspection or reporting requirements and authority, sunset or pre-emption provisions on this topic within the statute or passed separately; and exemptions. The codebook for the dataset will be adapted from the investigators’ previous work and represents the state of the art in public health law research and policy surveillance [25, 26].

Our survey of state laws uses the proprietary Westlaw legal database beginning with the following broad search terms applied to the entirety of the state statutory codes: [(influence intoxicat! drunk! alcohol) /50 firearm] and [(influence intoxicat! drunk! alcohol) /50 gun]. Legal research will begin with the most up-to-date full text versions of the statutes, then will follow the legislative history (where available) to construct a timeline for each law’s enactment date. For extreme risk protective order (ERPO) laws, we will use the following terms for our Westlaw search: “protective order” “protection order”, then do a keyword search of [(alcohol Intoxicat! drunk! Influence)] and review the results. Any substantive modifications of existing laws will be coded as a new event. Additional Westlaw research will be conducted to retrieve penalties and other relevant factors using keyword searches within our searches. For examples, see Table 1. We will compare our data to other public sources, such as the datasets previously mentioned and online data repositories such as Everytown USA, and assess and resolve any differences between them. Finally, should we be unable to locate citations in Westlaw, we will use LexisNexis or Hein Online to retrieve historical texts.

**Table 1:** Expected characteristics of alcohol-related firearm laws to be derived from legal statutes.

Factor	Description
Target	Who is affected and how is the target of the law is defined.
Alcohol	Does the law apply to acute intoxication, chronic intoxication, impairment, or some other definition? How exactly are intoxication, impairment, or influence (i.e. the terms used to describe someone who consumed alcohol in this context) legally defined?
Activity	Whether the law prohibits possession (which is legally broader in definition than carrying, covering circumstances such as having a firearm in the person’s car), both open and concealed carry, just concealed (or open) carry, and whether the law applies to guns that are loaded, unloaded, or both.
Type	Whether the law prohibits all firearms or just handguns or any other specific type.
Criteria for removal	Whether there are additional criteria defined as necessary before removal of the firearm, such as restaurant/bar owners having to provide explicit warning that firearms are not permitted on premise.
Sales	Who is held liable for violation of the law (buyer or seller)?
Duration	If, as a result of intoxication, a firearm license is denied or revoked or a gun is seized, for how long is the accused prohibited from purchasing, carrying, or possessing a firearm?
Violations	Which penalty level (felony or misdemeanor) is designated for violating the law?
Penalties	Is there mandated imprisonment (maximum and minimum mandated imprisonment period), mandated fines (maximum and minimum mandated fine amount) or any other penalties?
Exemptions	Are there any exemptions for certain individuals (e.g., law enforcement officers)?

Led by an experienced public health attorney on our team, each statute will be retrieved, reviewed and coded first by a team of law students, then by the attorney. Law students will be instructed on the protocol for retrieving the legal texts and will receive detailed training on translating the legal text into specific codes or variables. The training process includes checks on consistency of coding and, if new domains are identified through the textual analysis, the coding scheme will be updated and coders will be retrained on the revised protocol. The main coding will be performed by more than one individual with the supervising attorney serving as an arbitrator in the case of disagreement. All original legal citations will be included in the codebook and, in addition to the coded variables, detailed notes will be taken for each state law, especially in cases where the legal text is ambiguous or contradictory. The principal investigators will spot check variables and coding for consistency, review all coding protocols and training materials, and work alongside the attorney to develop a detailed codebook and well-organized dataset.

Two main analyses will be conducted on the resulting legal dataset. The first will focus on a binary indicator of the presence or absence of each of the different classes of alcohol-related firearm laws identified above. This includes a description of states with (and without) the four main categories of alcohol-related firearm laws to identify any patterns of law adoption by geography or time by developing a timeline of legal activity for each state. While this analysis simplifies the complexity of the laws themselves, it provides a means of assessing the impact of such laws on a range of related health outcomes. These binary data will be used to create a public use dataset with full documentation for deposit at a data repository such as ICPSR.

The second type of analysis focuses on the specific features of each law to describe their variation and to develop a multidimensional score for each state and year. For this content analyses, our team will develop a scoring system that assigns a maximum number of points based on the presence of specific provisions contained within the legal texts. While the final scheme will depend on the legal texts themselves, it will contain several components. First, the application of the law to different types of individuals (how clearly defined and inclusive is the definition of who is covered), the stringency of the law (the presence of exemptions for types of persons, locations, situations), the severity of violation (civil versus criminal, type of penalty, minimum and maximum penalty size/severity) and the potential enforceability of the law (level of clarity in defining the target population and assessing their level/type of alcohol involvement, presence of sentencing guidelines). Once preliminary coding is completed, a second attorney will perform spot checks of the coded data to review coding quality and consistency. Points for dimensions of strictness of each of the features identified in each law, as well as the presence of the law can then be summed, or operationalized as a percent of the total available points to create state-level summary scores for each state and year. Such scores will then be assessed empirically for their internal consistency and reliability.

## Discussion

This protocol describes the methods for identifying and coding US state-level alcohol-related firearm laws, and methods for operationalizing these laws to make them suitable for subsequent quantitative analyses. Our methods, used previously for developing a state policy score of over 33 alcohol laws [27], will be clearly documented and disseminated so that it can be updated in subsequent years by other researchers, allowing the dataset to be maintained over time. Given the long time period investigated, the measure can then be combined in quantitative models with other measures of laws, political economic and demographic characteristics, state resources and other measures to estimate whether such laws can be said to have an impact on a variety of health-related outcomes.

## References

[1] Kegler SR, Simon TR, Zwald ML, Chen MS, Mercy JA, Jones CM et al. Vital Signs: Changes in firearm homicide and suicide rates-United States, 2019–2020. MMWR Morb Mortal Wkly Rep. 2022; 71(19):656–63. doi: 10.15585/mmwr.mm7119e1 35550497 PMC9098246

[2] Xu JQ, Murphy SL, Kochanek KD et al. Deaths: Final data for 2016. Hyattsville, MD: National Center for Health Statistics, 2018 Contract No.:5. 30248015

[3] Branas CC, Han S, Wiebe DJ. Alcohol use and firearm violence. Epidemiol Rev. 2016; 38(1):32–45. doi: 10.1093/epirev/mxv010 26811427 PMC4762248

[4] Gani F. Sakran JV, Canner JK. Emergency department visits for firearm-related injuries in the United States, 2006–14. Health Aff (Millwood). 2017; 36(10):1729–38. doi: 10.1377/hlthaff.2017.0625 28971917

[5] Spitzer SA, Staudenmayer KL, Tennakoon L et al. Costs and Financial Burden of Initial Hospitalizations for Firearm Injuries in the United States, 2006–2014. Am J Public Health. 2017; 107(5):770–4. doi: 10.2105/AJPH.2017.303684 28323465 PMC5388949

[6] Everytown for Gun Safety. The economic cost of gun violence 2022. Accessed January 10, 2024: https://everytownresearch.org/report/the-economic-cost-of-gun-violence.

[7] Wintemute GJ. Alcohol misuse, firearm violence perpetration, and public policy in the United States. Prev Med. 2015, 79:15–21. doi: 10.1016/j.ypmed.2015.04.015 25937594

[8] Miller M, Hemenway. The relationship between firearms and suicide. A review of the literature. Aggress Violent Beh. 1999 4(1): 59–75. WOS:000076959400005.

[9] Hohl BC, Wiley S, Wiebe SJ, Culyba AJ, Drake R, Branas CC. Association of drug and alcohol use adolescent firearm homicide at individual, family, and neighborhood levels. JAMA Intern Med. 2017; 177(3): 317–24. 28055064 10.1001/jamainternmed.2016.8180 PMC5567686

[10] Choi NG, DiNotto DM, Sagna AO, Marti CN. Postmortem blood alcohol content among late-middle aged and older suicide decedents: associations with suicide precipitating/risk factors, means and other drug toxicology. Drug Alcohol Depend. 2018: (187)311–8. doi: 10.1016/j.drugalcdep.2018.02.034 29704853

[11] Anderson DM, Crost B, Dees DI. Wet laws, drinking establishments, and violent crime. The Economic Journal. 2018; 128(611): 1333–66.

[12] Kearns MC, Reidy DE, Valle LA. The role of alcohol policies in preventing intimate partner violence: a review of the literature. J Stud Alcohol Drugs. 2015; 17(1): 21–30. 25486390 PMC4770459

[13] Hedlund J, Forsman J, Sturup J, Masterman T. Pre-offense alcohol intake in homicide offenders and victims: a foresic-toxicological study. J Forensic Leg Med. 2018: 56:55–58. . 29533206 10.1016/j.jflm.2018.03.004

[14] Borges G, Bagge CL, Cherpitel CJ et al. A meta-analysis of acute use of alcohol and the risk of suicide attempt. Psychol Med. 2017; 47(5):949–57. doi: 10.1017/S0033291716002841 27928972 PMC5340592

[15] Park CHK, Yoo SH, Lee J et al. Impact of acute alcohol consumption on lethality of suicide methods. Compr Psychiatry. 2017; 75:27–34. doi: 10.1016/j.comppsych.2017.02.012 28288368

[16] Naimi TS, Xuan Z, Coleman SM et al. Alcohol policies and alcohol-related policies and alcohol-involved homicide victimization in the United States. J Stud Alcohol Drugs. 2017; 78(5): 781–8. 28930066 10.15288/jsad.2017.78.781 PMC5675429

[17] Xuan Z, Naimi TS, Kaplan MS et al. Alcohol policies and suicide: a review of the literature. Alcohol Clin Exp Res. 2016; 40(10): 2043–55. doi: 10.1111/acer.13203 27618526 PMC5048547

[18] Trangenstein PJ, Curriero FC, Jennings JM et al. Methods for evaluating the association between alcohol outlet density and violent crime. Alcohol Clin Exp Res.2019; 43(8):1714–26. doi: 10.1111/acer.14119 31157919 PMC6679806

[19] Trangenstein PJ, Eck RH, Lu Y et al. The violence prevention potential of reducing alcohol outlet access in Baltimore, Maryland. J Stud Alcohol Drugs. 2020; 81(1):24–33. doi: 10.15288/jsad.2020.81.24 32048598 PMC7024813

[20] Trangenstein PJ, Greene N, Eck RH et al. Alcohol advertising and violence. Am J Prev Med. 2020; 58(3): 343–51. doi: 10.1016/j.amepre.2019.10.024 31980304 PMC7140760

[21] Xuan Z, Blanchette J, Nelson TF et al. The alcohol policy environment and policy subgroups as predictors of binge drinking measures among US adults. Am J Pub Health. 2015; 105(4): 816–22. WOS00035738780005. doi: 10.2105/AJPH.2014.302112 25122017 PMC4358175

[22] Carr BG, Porat G, Wiebe DJ, Branas CC. A review of legislation restricting the intersection of firearms and alcohol in the U.S. Pub Health Rep. 2010; 125(5): 674–9. doi: 10.1177/003335491012500509 20873283 PMC2925003

[23] RAND State Firearm Law Database. 2018. https://www.rand.org/research/gun-policy/law-navigator.html

[24] State firearms laws database. 2020. https://mail.statefirearmlaws.org/state-state-firearm-law-data

[25] Burris S, Ashe M, Levin D et al. A transdisciplinary approach to public health law: the emerging practice of legal epidemiology. Annu Rev Public Health. 2016; 37:135–48. doi: 10.1146/annurev-publhealth-032315-021841 26667606 PMC5703193

[26] Burris S, Hitchcock L, Ibrahim J et al. Policy surveillance: a vital public health practice comes of age. J Health Polit Policy Law. 2016; 41(6):1151–1173. doi: 10.1215/03616878-3665931 27531941

[27] Silver D, Macinko J, Giorgio M, Bae JY. Evaluating the relationship between binge drinking rates and a replicable measure of U.S. alcohol policy environments. PLos One.2019; 14(6):e0218718. . 31237888 10.1371/journal.pone.0218718 PMC6592603

